# Self-healing photo-neuropathy and cervical spinal arthrosis in four sisters with brachioradial pruritus

**DOI:** 10.1186/1749-7221-4-21

**Published:** 2009-11-17

**Authors:** Joanna Wallengren

**Affiliations:** 1 Department of Dermatology, University Hospital, Lund, Sweden

## Abstract

The cause of brachioradial pruritus (a localized itching on the arms or shoulders) is controversial. The role of sun and cervical spine disease has been discussed. This is a report on four sisters suffering from brachioradial pruritus recurring every summer. The sisters spent much time outdoors and exposed themselves extensively to the sun. They also had occupations requiring heavy lifting. Cervical radiographs indicated arthrosis. The density of sensory nerve fibers in the skin biopsies from the itchy skin of the arms, visualized by antibodies against a pan-neuronal marker, protein gene product 9.5, was reduced compared with biopsies from the same skin region during the symptom-free period in the winter. This data exemplifies that brachioradial pruritus is a self healing photoneuropathy occurring in middle aged adults predisposed by cervical arthrosis.

## Letter

"Solar pruritus of the elbows or brachioradial summer pruritus," a localized itch of the skin on the dorso-lateral aspect of the arm, was first described by Waisman in Florida 1968 [1]. Walcyk and Elpern, who described 42 Hawaian patients with chronic intermittent pruritus, suggested brachioradial pruritus to be a photo-neurological disorder caused by sun-induced damage to nerve endings that results in pruritus and altered sensation in susceptible individuals [2]. Since, several patients from temperate zones showing seasonal occurrence of brachioradial pruritus have been described [3-9].

Another hypothesis concerning etiology of brachioradial pruritus was presented by Heyl in South Africa, who suggested that this disorder may be caused by nerve injury to the cervical spine or by nerve compression at other locations because 5 out of his 14 patients had a history of neck trauma or arthritis [10]. In favour of this hypothesis is a report on 22 patients with brachioradial pruritus of whom 11 had cervical spine radiographs showing pathological changes correlating with the location of pruritus in each of these 11 patients [11]. In the Hawaiian patients of Walcyk and Elpern, radiographs of 15 patients showed changes only in the older, arthritis-age groups [2].

The present report concerns investigation of the density of the sensory nerve fibers in skin biopsies taken from the affected skin in the itchy period and in the symptomless period as well as radiography of cervical spine in four sisters with brachioradial pruritus. The pedigree of the three generations of the family of the sisters as well as the radiological findings of the cervical spine in these patients has been reported previously [12].

In the present study skin biopsy specimens from itchy skin were collected in October and were compared with biopsies from adjacent skin collected in March when the patients had no itch. The cutaneous innervation was visualized by antibodies against a pan-neuronal marker, protein gene product 9.5 [13]. The nerve fibers were counted in three sections of each biopsy, the mean being presented in Table 1 which also summarizes the clinical data of the patients.

**Table 1:** Summary of the clinical and experimental data of the four sisters.

Pat	History	Profession	Habits	Clinical findings	Nerve density	Radiography
1/59 y	Recurrent severe itch on the radial aspect of the lower arms appearing in August lasting to December for 12 y, neck pain	Clerk	Out-door activites	Normal appearing skin on the lower arms, hypoesthesia to pinprick	79 ± 11/256 ± 55	Arthrosis of the uncovertebral joint C5

2/73 y	Recurrent itch on the lateral aspect of the upper arms for 28 y, neck pain	Hostess of a school kitchen	Out-door activites	Normal appearing skin on the upper arms	211 ± 39/264 ± 29	Arthrosis of the intervertebral joint C7 and severe arthrosis of the uncovertebral joints C5-C6, with narrowing of the foramina

3/71 y	Recurrent itch on the lateral aspect of the upper arms for 26 y, neck pain	Shop-keeper	Out-door activites	Normal appearing skin on the upper arms	333 ± 76/163 ± 38	Arthrosis of the uncovertebral joints C5-C6 and a reduction in the height of disc C6

4/67 y	Recurrent severe itch on the radial aspect of the lower arms for 13 y, neck pain	Nurse	Out-door activites	Normal appearing skin on the lower arms	159 ± 26/429 ± 5	Severe arthrosis of the uncovertebral joints C5-C6, with narrowing of the foramina, a prominent reduction in the height of discs C5-C6, a severe arthrosis of intervertebral joint C7 and compression of vertebra C5.

The biopsies taken in October revealed a lower density of PGP 9.5 immunoreactive nerve fibers compared with the control biopsy taken in March when the patients had no itch (195 ± 104 vs 264 ± 99), which has been described previously [14]. The same phenomenon of loss of epidermal and dermal nerve fibers has been shown following phototherapy [15]. It seems that the number of cutaneous nerve fibers is lowered in itchy skin of patients with brachioradial pruritus but normalizes after recovery suggesting that this type of photo-neuropathy is self-healing.

All our patients reported neck pain which may be due to their professions, since they all were occupied with heavy lifting. It seems that brachioradial pruritus appearing at the end of each summer occurred first at the age of about 45 suggesting that the age of the patients is also of importance. Cervical radiography of two of our patients (patient 2 and 4) displayed a narrowing of foramina between the fifth and sixth cervical vertebral bodies, which could result in a nerve root impingement. Narrowing of foramina is most common at this level of the cervical column, being demonstrated in 22% of 160 asymptomatic individuals between thirty and seventy years of age [16]. Radiography of the cervical spine is a crude method, correlating poorly with clinical dysfunction or pain. With aging, degenerative changes increase in the cervical column, occurring in about 75% of asymptomatic individuals at age of 60-70 years [16]. The only definitive diagnostic means of determining nerve root impingement currently available is MRI, which has been performed on only a few of the published cases of brachioradial pruritus, one of whom had a spinal cord tumor which led to brachioradial pruritus involving the C5-C6 dermatomes [17-20]. Cervical spine disease is normally a permanent disorder, and one would expect continuous neuropathic pain or itch as a consequence of it. Spinal disease alone cannot explain the symptoms of brachioradial pruritus, which in our patients was characterized by symptom-free periods broken off by relapse late in the summer each year. In my opinion, the data presented suggests that brachioradial pruritus is a self healing phototherapy occurring in middle aged adults predisposed by cervical arthrosis [21]. What is your opinion?
